# Discrete element modeling and experimental study of biomechanical properties of cotton stalks in machine-harvested film-stalk mixtures

**DOI:** 10.1038/s41598-024-62390-8

**Published:** 2024-06-05

**Authors:** Jia Zhang, Jianhua Xie, Yakun Du, Yuanze Li, Yong Yue, Silin Cao

**Affiliations:** 1 College of Mechanical and Electrical Engineering, Xinjiang Agricultural University, Urumqi, 830052 China; 2 College of Mechanical and Electrical Engineering, Xinjiang Institute of Engineering, Urumqi, 830023 China; 3 Xinjiang Key Laboratory of Intelligent Agricultural Equipment, Urumqi, 830052 China

**Keywords:** Materials science, Biomaterials, Information theory and computation

## Abstract

To address the current problems of low accuracy and poor reliability of the discrete element model of cotton stalks, as well as the difficulty of guiding the design and optimization of the equipment through simulations, the discrete element modeling and physical-mechanical tests of cotton stalks in machine harvested film-stalk mixtures are carried out. The peak tensile force $F_{\rm j}^{\max }$, the peak pressure $F_{\rm y}^{\max }$, the peak bending force $F_{\rm w}^{\max }$, the peak shear force $F_{\rm j}^{\max }$, and the force-displacement (*F*–*x*) curves of cotton stalks are obtained from the physical tests. The discrete element model of double-layer cotton stalks based on the flat-joint model is established with the PFC$^{\rm 3D}$ software. The $F_{\rm y}^{\max }$ is taken as the response value, and the microscopic parameters of the cotton stalk model are used as the test factors, then the Plackett–Burman test, the steepest climb test, and the Box–Behnken test are sequentially designed using Design-Expert software. The second-order regression model describing the relationship between the $F_{\rm y}^{\max }$ and the microscopic parameters is established. The optimal parameter combinations of the microscopic parameters are obtained, and then they are utilized to construct the compression, bending, and shear models of cotton stalks and to carry out the validation tests. The results confirm that the established discrete element model could accurately characterize the biomechanical properties of cotton stalks and that the parameter calibration method is reasonable, which could provide a reference for the discrete element modeling of cotton stalks and other stalks, and also offer a theoretical basis for the research of the crushing and separation mechanism of the film-stalk mixtures and the development of the equipment.

## Introduction

As of 2022, the crop area in Xinjiang, China alone is 2.40 million hectares, accounting for more than 70% of the total sown area. The total weight of film used each year is about 170,000 tons, which is easy to retain in the farmland soil after use, with a greater impact on soil properties, crop growth, and surrounding environment^1,2^. Existing residual film recycling machines can solve the problem of film residue in cotton fields to a large extent^3^. Most of the film-stalk mixtures recovered by residual film recycling machines are still burned or landfilled, resulting in secondary environmental pollution^4^.

The film-stalk mixtures recovered by the residual film-recycling machine mainly consist of cotton stalks, used mulch, and soil impurities^5^. The cotton stalks can be made into animal feed, fuel, and adsorbent materials, while used mulch can be washed to make products such as plastics and drip irrigation tapes^6^.However, the resourceful reuse of used mulch or cotton stalks requires first chopping the mulch mixture and separating the used protection from the cotton stalks^7,8^. Due to the characteristics of cotton stalks with high hardness, many fibers, and low water content, their structure and mechanical properties are quite different from those of other crop stalks^9^, which leads to the problems of uneven stalk chopping and high chopping resistance of film-stalk mixtures in the chopping process and seriously restricts the application and promotion of the mechanized chopping and separating technology of the film-stalk mixtures. Therefore, the analysis of the biomechanical properties of cotton stalks in the mixture is of great significance in studying the chopping mechanism of film-stalk mixtures and the development of chopping equipment.

With the rapid development of computer simulation technique, the research method of biomechanical properties of plant stalks has been transitioned from the traditional physical test method to the combination of physical test and computer simulation test^10,11^. This new method can realize the in-depth analysis of the micromechanical properties, deformation, and damage mechanisms of plant stalks, and then explore the macroscopic mechanical properties of plant stalks. Compared with the traditional physical test methods, the numerical simulation methods based on discrete element simulation have the advantages of high efficiency, reliable results, and repeatability^12^. In recent years, the discrete element method has been widely applied in the field of agricultural machinery. Wang et al.^12^ constructed a discrete unit model of citrus fruit stalks by EDEM simulation software and calibrated the accurate discrete element parameters to efficiently simulate the bending and shearing process of citrus stalks^13^. Sadrmanesh and Chen^14^ used PFC$^{\rm 3D}$ software to establish a discrete element model of cannabis stalks and the discrete element parameters were calibrated with the results of physical tests, which can effectively simulate the tensile behavior of cannabis stalks^14^. Liao et al.^15^. used EDEM simulation software to establish a discrete element simulation model of fodder rape stalks and investigated the effects of determined discrete element parameters on the mechanical properties of fodder rape stalks^15^. Liu et al.^16^ used EDEM simulation software to establish a two-layer discrete element model of corn stover, and the mechanical parameters of the outer skin and inner flesh of corn stalks were calibrated^16^. Guo et al.^17^ established a mechanical model of banana stalks by combining physical tests and discrete element simulation to study the shear, tensile, compression, and bending mechanical properties of banana stalks with different banana varieties and internode positions, and analyzed the microscopic mechanical change rules of banana stalks and their nonlinear damage behaviors from a microscopic point of view^17^. In addition, the discrete element method has also been applied in the analysis of materials such as rice stalks^18,19^, wheat stalks^16,20^, and caraway stalks^21^. The above studies have demonstrated that the discrete element method has become an important method for simulating plant stalks. Currently, the discrete element software mainly includes EDEM software and PFC$^{\rm 3D}$ software, and the PFC$^{\rm 3D}$ software can be used not only for modeling bulk flow behavior and mechanical analysis of mixed materials^22^, but also for describing the microscopic or macroscopic crack changes and damage deformation states^23^. Since it does not need to take into account whether the medium is continuous or not and whether there is a transition from continuous to discrete in the medium, it has a great advantage in the study of discrete problems of continuous materials, such as the deformation, damage, and fracture of plant straw, and the local mechanical behaviors inside the model^17,24^.

However, there are fewer studies on the biomechanical properties of cotton stalks in machine-harvested film-stalk mixtures, as well as the nonlinear damage mechanics and deformation damage mechanism. There is still a gap in the micromechanical modeling and simulation analysis of the force deformation process of cotton stalks in machine-harvested film-stalk mixtures. To elucidate the micro-mechanical damage mechanism and the evolution of mechanical property parameters of cotton stalks, and to further provide a basis for the research on the crushing mechanism of film-stalk mixtures as well as the design and development of crushing equipment, this paper takes the cotton stalks in the film-stalk mixtures as the research object, and adopts a combination of simulation test and physical test to investigate the bio-mechanical characteristics of cotton stalks. The discrete element models of mechanical properties of cotton stalks in tension, compression, bending, and shear scenarios are established by using PFC$^{3D}$ simulation software, and the microscopic parameters of the cotton stalks are calibrated according to the physical test results. The current study provides a theoretical basis for the analysis of the crushing mechanism of the film-stalk mixtures, the design of crushing equipment, as well as the discrete element modeling, and the biomechanical characterization of the other stalk materials.

## Materials and methods

### Materials

**Figure 1:**
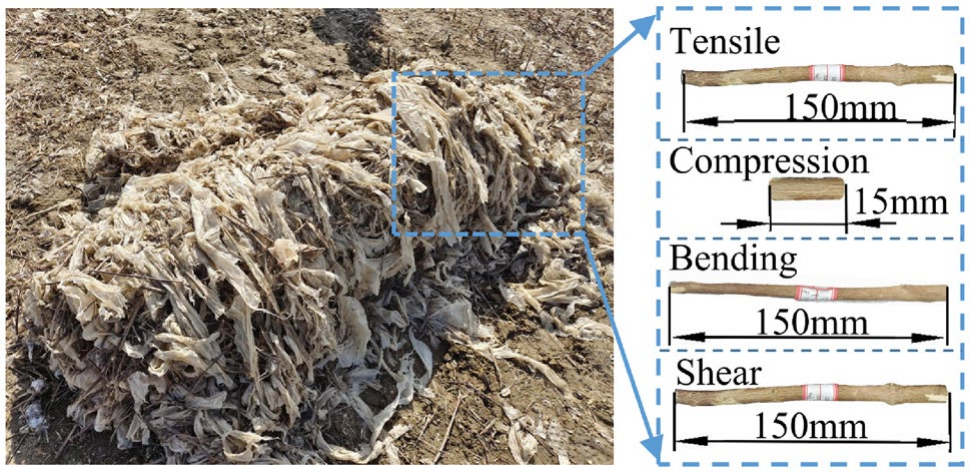
Selection of cotton stalk samples in residual film mixture.

Samples of the film-stalk mixture used in the tests were taken in April 2023 from Maras County, Xinjiang, China, as shown in Fig. 1. It is determined that the film-stalk mixture mainly consists of cotton stalks (about 35.5%), residual film (about 21.8%), and soil particles (about 42.7%). Relatively intact and unbroken cotton stalks are selected from the film-stalk mixture, and then the middle and lower parts of the selected stalks are cut into long cotton stalks with a length of 150 mm (for tensile, bending, and shear tests) and short cotton stalks with a length of 15 mm (for ring compression test). To ensure the accuracy of the data, cotton stalks with small differences in diameter are selected for sampling as much as possible, and 10 stalks are taken for each test, with an average diameter of $7.49\pm 0.21$ mm, an average density of 727.9 kg/m$^3$, and an average water content of 4.83%.

### Mechanical properties tests on cotton stalks

**Figure 2:**
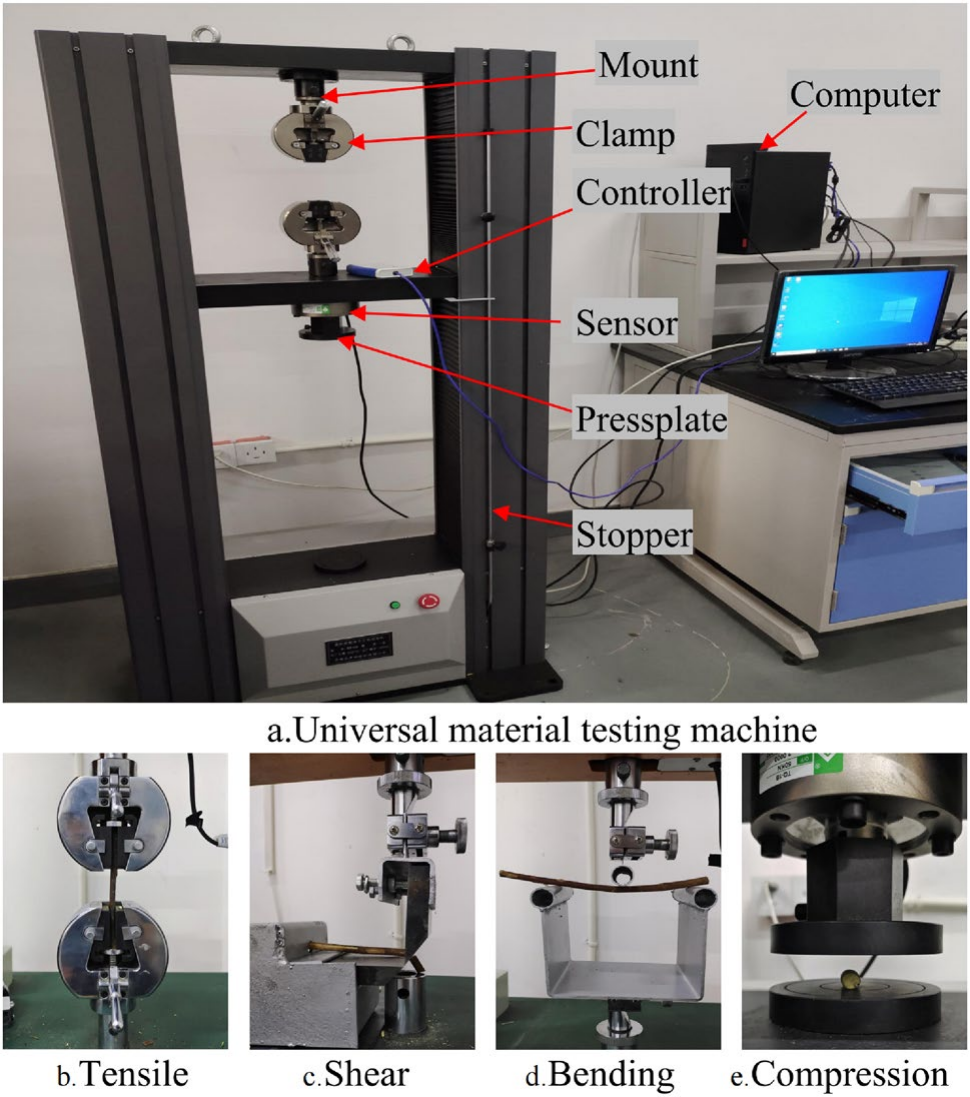
Physical and mechanical tests of cotton stalks.

As presented in Fig. 2a, a universal testing machine (WDW-50, Jinan Yongduo Test Press Co., Ltd., China) is used to perform tensile, compression, three-point bending, and shear tests on the cotton stalks to obtain the $F_{\rm l}^{\max }$, $F_{\rm y}^{\max }$, $F_{\rm W}^{\max }$, and $F_{\rm j}^{\max }$, which are then utilized to determine the tensile strength $\sigma _{\rm l}^{max}$, compressive strength $\sigma _{\rm y}^{\max }$, bending strength $\sigma _{\rm w}^{\max }$, and shear strength $\sigma _{\rm j}^{\max }$ of the cotton stalks.

#### Tensile test

The tensile mechanical properties of the cotton stalks are clarified by tensile tests. The test procedure is displayed in Fig. 2b, where both ends of the cotton stalks are fixed in the upper and lower fixtures, and the loading rate is 5.0 mm/min. A total of 10 tests are conducted, and the test results are averaged. The peak tensile force when the cotton stalks are fractured is recorded as $F_{\rm l}^{\max }$, and the tensile strength of the cotton stalks $\sigma _{\rm l}^{max}$ is calculated by Eq. (1)^17^:

1 $$\begin{aligned} {\sigma _1}^{\max }=\frac{F_1^{\max }}{A_{\rm m}} \end{aligned}$$

where $F_{\rm l}^{\max }$ is the peak tensile force, with a unit of N, and $A_{\rm m}$ is the cross-sectional area of the cotton stalk, with a unit of m$^2$.

#### Ring compression test

The compressive mechanical properties of cotton stalks are clarified by the ring compression test, as shown in Fig. 2e. The cotton stalks are placed between the upper and lower platens, in which the spacing of the upper and lower parallel platens is 100 mm, and the loading rate is 5.0 mm/min. A total of 10 tests are conducted, and the test results are averaged. The $F_{\rm y}^{\max }$ when the cotton stalks are broken is recorded, and the ring compressive strength $\sigma _{\rm y}^{max}$ of cotton stalks is calculated by Eq. (2):

2 $$\begin{aligned} {\sigma _y}^{\max }=\frac{F_y^{\max }}{L_{\rm m}D_{\rm m}} \end{aligned}$$

where $F_{\rm y}^{\max }$ indicates the peak force, with a unit of N, $D_{\rm m}$ is the outer diameter of the stalk to be compressed, with a unit of m, and $L_{\rm m}$ is the length of the stalk to be compressed, with a unit of m.

#### Three-point bending test

The three-point bending test was used to clarify the bending mechanical properties of cotton stalks. As shown in Fig. 2d, the test fixture is a homemade fixture including an upper-pressure tube and two lower support tubes. The diameters of the lower support tubes and the upper-pressure tube are 20 mm, and the distance between the two lower support tubes is 120 mm. The two support pedestals are fixed on the workbench of the testing machine, and the cotton stalks are placed smoothly on the two lower support tubes. The upper-pressure tube is adjusted to the position close to the cotton stalks, and the loading rate is set to 5.0 mm/min. A total of 10 tests are carried out, and the test results are averaged. The peak bending force $F_{\rm W}^{\max }$ is recorded when the cotton stalks break. The $\sigma _{\rm w}^{\max }$ of the cotton stalks is calculated by Eq. (3)^13^:

3 $$\begin{aligned} \sigma _\mathrm{{w}}^{\max }\mathrm{{ = }}\frac{{8{F_\mathrm{{w}}}^{\max }P}}{{\pi {D_\mathrm{{m}}}^3}} \end{aligned}$$

where $F_{\rm W}^{\max }$ denotes the peak bending force (unit of N), *P* is the spacing between the lower support tubes (unit of m), and $D_{\rm m}$ is the diameter of the cotton stalk sample (unit of m).

#### Shear test

The shear mechanical properties of cotton stalks are clarified through the shear tests. The operating principle of the chopper homemade shear fixture of the film stalk mixture is presented in Fig. 2c. The shear fixture includes a movable knife and a fixed knife, and the angle between the movable knife and the fixed knife is 90$^\circ$. The cotton stalks are fixed horizontally on the fixed knife to form a single-support cutting. The distance between the movable knife and the fixed knife is 0.1 mm. In the shear test, the movable knife is loaded at a loading rate of 5 mm/min. A total of 10 tests are carried out, and the results are averaged. The $F_{\rm j}^{\max }$ is recorded when the cutting of the cotton stalks occurs, and the $\sigma _{\rm j}^{\max }$ of the cotton stalks is calculated by Eq. (4)^25^:

4 $$\begin{aligned} \sigma _\mathrm{{j}}^{\max }\mathrm{{ = }}\frac{{{F_\mathrm{{j}}}^{\max }}}{{2{A_\mathrm{{m}}}}} \end{aligned}$$

where $F_{\rm j}^{\max }$ is the peak shear force (unit of N), and $A_{\rm m}$ is the cross-sectional area of the cotton stalk sample (unit of m$^2$).

### DEM of cotton stalk

#### Modeling the bilayer flexibility of cotton stalk

**Figure 3:**
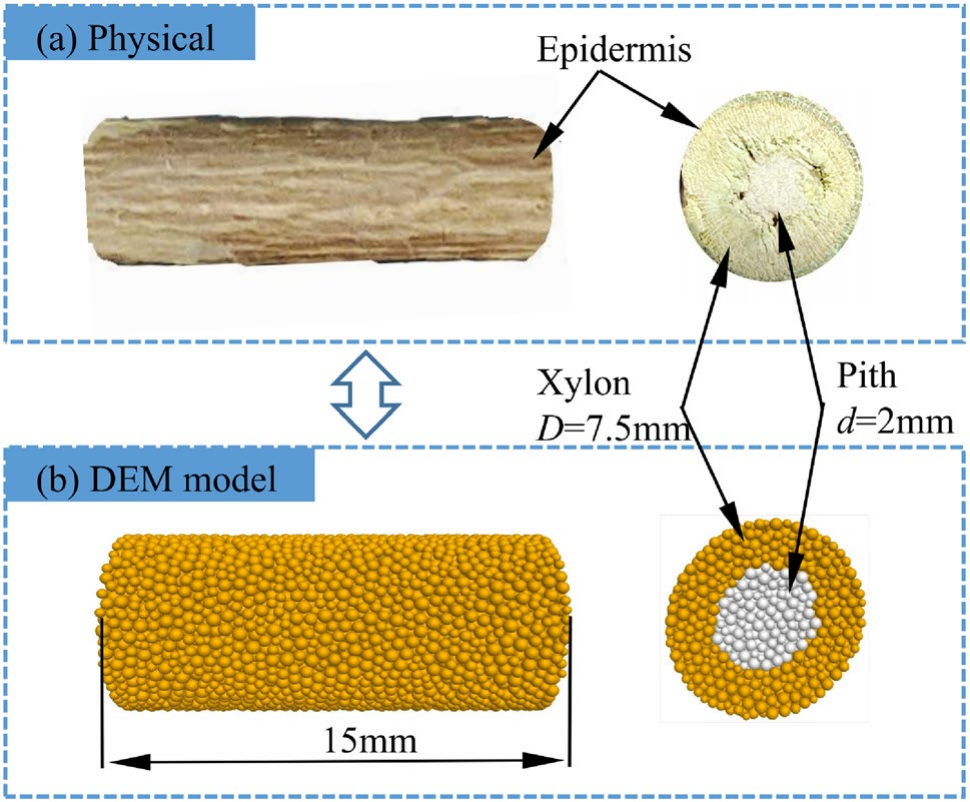
Physical model and simulation model of cotton stalk.

As revealed in Fig. 3a, analyzing the characteristics of the cotton stalk cross-section in the film-stalk mixture identifies that the cotton stalks consist of three components epidermis, xylem, and pith core. The cotton stalk has a thin epidermis and weak mechanical properties, so the epidermis and xylem can be simplified into a physical model. The pith core differs significantly from the epidermis and the xylem in terms of material properties^26^, with the xylem being greater than the pith core in both density and strength. The average diameter of cotton stalks in the film-stalk mixture is $7.49\pm 0.21$ mm, with an average diameter of $2.08 \pm 0.11$ mm in the pith core.

The procedure for developing a simulated compression model of cotton stalk is as follows. (1) Establish a wall with a diameter of 7.5 mm and a length of 15 mm, and generate basic ball particles within the wall. (2) Perform structural subdivision using the spatial location of the ball particles to generate polygonal aggregation regions. (3) Delete the basic ball particles to generate a uniformly distributed particle aggregate within a particle size range of 0.15–0.25 mm. Servo the model to an equilibrium state for releasing stresses between particles. (4) All particles are grouped according to the region in which they are located, and the region with the particle diameter of 2.0 mm is defined as the pith model, and the region outside the pith model is defined as the xylem model, i.e., the dimensions of the pith model are 2.0 mm in diameter and 15.0 mm in length, and the dimensions of the xylem model are 7.5 mm in diameter of the outer circle, 2.0 mm in diameter of the inner circle, and 15.0 mm in length. (5) The cotton stalk bilayer flexibly model is generated by filling the particles of the xylem model, the particles of the pith core model, and the boundaries of the xylem and pith cores models, respectively, with different adhesive bond parameters.

All the above model details are compiled using the built-in FISH language of the PFC3D software. By preparing the modeling program, a simulated compression model of cotton stalks is finally established, as shown in Fig. 3b. There are 12,026 particles in the cotton stalks model, of which 8614 particles are in the xylem model and 3412 particles are in the pith model. The total number of bonds in the cotton stalks model is 58,403, of which 39,029 are in the xylem model, 15,703 in the pith model, and 3671 between the xylem and pith models.

#### Flat-joint model

**Figure 4:**
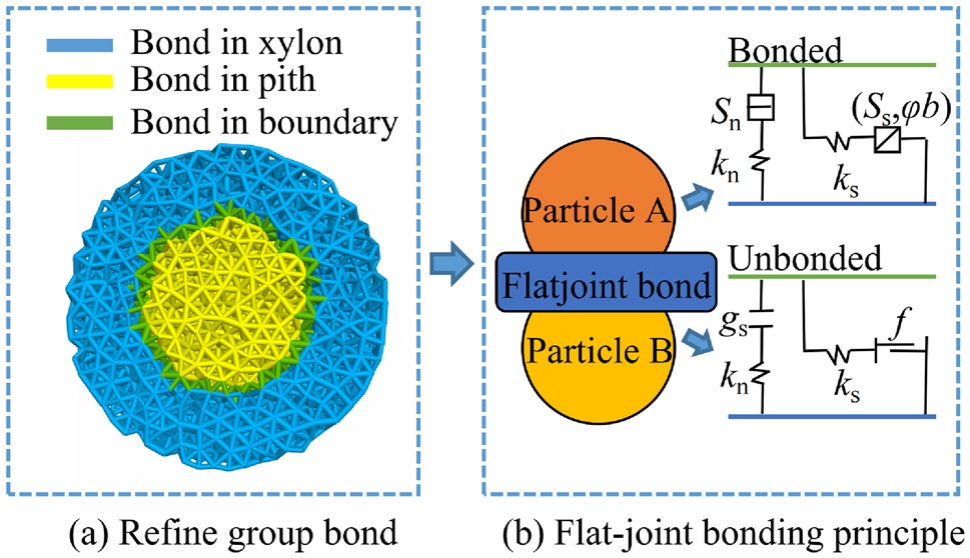
Bond distribution and bond principle.

The flat-joint model in PFC$^{\rm 3D}$ is utilized to simulate the gluing state of the internal tissues of cotton stalks. As indicated in Fig. 4a, the bond of particles within the cotton stalk model mainly consists of three types: particle bonding within the xylem, particle bonding within the pith core, and particle bonding at the boundary between the xylem and the pith core. To simplify the analysis, the above three types of bonds are added to the flat-joint model. As indicated in Fig. 4b,The flat-joint model could be simplified as a set of “spring” elements with constant normal stiffness and tangential stiffness, and “sticky pot” elements with certain tensile and compressive capabilities^27^. The flat-joint model uses a flat surface to bond two particles, and the bonding surface consists of segmented units^24^. Each unit has bonded and unbonded modes. When the loaded unit on the bonding surface reaches the critical strength of bonding, the bonded unit will be destroyed and become a non-bonded unit.

### Determination of parameters and range of discrete element model for cotton stalk

To simulate the macroscopic mechanical properties of cotton stalk, it is necessary to determine the microscopic parameters of the particles and contacts^28^ in the flat-joint model^29^. The microscopic parameters of particles include particle normal stiffness $K_{n\mathrm b}$, wall-normal stiffness $K_{\rm w}$, particle contact modulus *E*, particle contact stiffness ratio $k_{\rm g}$, particle friction factor $\mu _{\rm b}$, particle density $\rho$, minimum particle radius $R_{\rm min}$, particle size ratio $R_{\max }/R_{\rm min}$. The microscopic parameters of flat-joint contact include flat contact modulus E*, normal bonding strength $\sigma _{\rm n}$, tangential bonding strength $\sigma _{\rm s}$ flat nodal stiffness ratio $k_{\rm g}$*, straight joint friction factor $\mu _{\rm c}$, straight joint cohesion $C_{\rm b}$, installation gap ratio $g_{\rm r}$, straight joint internal friction angle $\varphi _{\rm b}$, number of radial units $N_{\rm r}$, number of circumferential units $N_{\rm a}$, radius coefficient $\lambda$. To minimize the number of calibrations, the following assumptions are made based on the results of previous research^30^. Setting the same values for microscopic parameters in flat-joint models with the same significance::$E_{\rm b}$=$E_{\rm c}$, $k_{\rm g}$=$k_{\rm g}$*. Based on the test results and the findings in the available literature, the microscopic parameters that have little effect on the macroscopic parameters are determined as follows: $\rho$=727.9 kg/m$^3$, $R_{\rm min}$=0.15 mm, $R_{\max }/R_{\rm min}$=1.66, $\lambda$=1, $\varphi _{\rm b}$=0, $g_{\rm r}$=0.32, $N_{\rm r}$=2, and $N_{\rm a}$=4.

To obtain the microscopic parameters of the cotton stalk model more accurately, a double-layer model of cotton stalk, i.e., a xylem model and a pith core model, is established by PFC$^{\rm 3D}$ software. Therefore, the microscopic parameters of cotton stalk include xylem-xylem, pith-pith, and xylem-pith. Combining the above assumptions, the parameters of the cotton stalk are simplified as particle normal stiffness $K_{n\mathrm b}$, wall-normal stiffness $K_{\rm W}$, stiffness ratio $k_{\rm g}$, bond modulus (xylem-xylem) *E*(x-x), normal bond strength (xylem-xylem) $\sigma _{\rm n}$(x-x), tangential bond strength (xylem-xylem) $\sigma _{\rm s}$(x-x), bond modulus (pith-pith) *E*(p-p), normal bond strength (pith-pith) $\sigma _{\rm n}$(p-p), tangential bond strength (pith-pith) $\sigma _{\rm s}$(p-p), bond modulus (xylem-pith) *E*(x-p), normal bond strength (xylem-pith) $\sigma _{\rm n}$(x-p), tangential bond strength (xylem-pith) $\sigma _{\rm s}$(x-p) and the coefficient of bond friction, $\mu _{\rm c}$. Although the above parameters can be derived experimentally, it is difficult to obtain their accurate values due to the large differences in the size and shape of the sampled cotton stalk. By referring to the results of related stalk simulation studies^12,13,31^. The initial range of approximate values of the parameters to be calibrated for the discrete elemental model of cotton stalk is determined, as shown in Table 1.

**Table 1:** *PBD* test factor codes.

Type		Symbol.	Parameter levels		
		Parameter	Low (− 1)	Medium (0)	High (1)
Linear parameters		X1:$K_{n{\rm b}}$/(N/m)	1.0 × 10$^6$	1.0 × 10$^7$	1.0 × 10$^8$
	Particle parameters	X2:$K_\mathrm{{w}}$(N/m)	1.0 × 10$^7$	1.0 × 10$^8$	1.0 × 10$^9$
		X3:$k_\mathrm{{g}}$	1.0	1.5	2.0
	Xylem-Xylem	X4:*E*(x-x)/(Pa)	5.0 × 10$^7$	1.0 × 10$^8$	5 × 10$^8$
		X5:$\sigma _{\rm n}$(x-x)/(Pa)	1.0 × 10$^6$	5.0 × 10$^6$	1.0 × 10$^7$
	Pith-Pith	X6:$\sigma _{\rm s}$(x-x)/(Pa)	5.0 × 10$^6$	1.0 × 10$^7$	5.0 × 10$^6$
Flat-joint bonding parameters		X7:*E*(p-p)/(Pa)	5.0 × 10$^5$	1.0 × 10$^6$	5.0 × 10$^6$
		X8:$\sigma _{\rm n}$(p-p)/(Pa)	1.0 × 10$^4$	5.0 × 10$^4$	1.0 × 10$^5$
		X9:$\sigma _{\rm s}$(p-p)/(Pa)	5.0 × 10$^4$	1.0 × 10$^5$	5.0 × 10$^5$
	Xylem-pith	X10:*E*(x-p)/(Pa)	1.0 × 10$^8$	5.0 × 10$^8$	1.0 × 10$^9$
		X11:$\sigma _{\rm n}$(x-p)/(Pa)	1.0 × 10$^7$	5.0 × 10$^7$	1.0 × 10$^8$
		X12:$\sigma _{\rm s}$(x-p)/(Pa)	1.0 × 10$^7$	5.0 × 10$^7$	1.0 × 10$^8$
	Friction coefficient	X13:$\mu _{\rm c}$	0.2	0.5	0.8

### Parameter calibration method

The physico-mechanical tests of cotton stalks are performed with the universal material machine presented in Fig. 2, and the $F_{\rm l}^{max}$, $F_{\rm y}^{max}$, $F_{\rm W}^{max}$, $F_{\rm j}^{max}$, and *F*–*x* curves of cotton stalks are obtained.

The ring compression test of cotton stalk demonstrates that the inner side of the stalk is subjected to radial compression and the outer side is subjected to tension. When the tension reaches the peak, the extrusion occurs, so the ring compression test is selected for the simulation calibration test. First, with $F_{\rm y}^{\max }$ as the response value, the Plackett-Burman test method (*PBD* test) is used to screen the significant factors affecting the $F_{\rm y}^{\max }$ of cotton stalks. Then the steepest climb test is carried out to determine the optimal range of the significant influence factors. Finally, the Box-Behnken test method (*BBD* test)^24^ is used to establish a regression model describing the relationship between the significant influence factors and $F_{\rm y}^{\max }$. The regression model is solved to obtain the optimal combination of parameters affecting $F_{\rm y}^{\max }$. In addition, the simulated bend and shear models of cotton stalks are established to validate the accuracy of microscopic parameters of the cotton stalks, where the optimal combination of parameters obtained from the simulated compression test is used.

#### *PBD* test

With 13 microscopic parameters determined and with test factors and $F_{\rm y}^{\max }$ as the evaluation index, Design-Expert software is applied to simulate the *PBD* test to screen out the factors that have a significant influence on the $F_{\rm y}^{\max }$. To explore the influence law, based on several pre-simulation tests and simulation parameters of the stalk materials, the value range of the test factors is set to 2 center points, and a total of 20 groups of tests are carried out. The value range of microscopic parameters of cotton stalks is shown in Table 1, and the test results are illustrated in Table 3.

#### Steepest climb test

Based on the results of the *PBD* test, the steepest climb test is conducted for the microscopic parameters to be calibrated, and the effects of these parameters on the *F*–*x* curve are investigated. The relative error $\delta$ between the peak pressure $F_{\rm y}^{\max }$ of the simulation test and the average peak pressure $F_{\rm ya}^{\max }$ of the physical test is also examined.The methodology and the test results of the steepest ascent test are given in Table 5, in which the relative error $\delta$ is determined by Eq. (5):

5 $$\begin{aligned} \delta \mathrm{{ = }}\frac{{\left| {{F_y}^{max}\mathrm{{ - }}{F_{y\mathrm{{a}}}}^{max}} \right| }}{{{F_{y\mathrm{{a}}}}^{max}}} \end{aligned}$$

where $F_{\rm y}^{\max }$ is the peak force, and $F_{\rm ya}^{\max }$ is the average value of the peak force in the physical test (with a unit of N).

#### BBD test

Based on the results of *PBD* test and Steepest ascen test, a four-factor three-level *BBD* test is carried out, as shown in Table 6. The results of the test are utilized to establish a second-order regression model for the peak pressure $F_{\rm y}^{\max }$. The average peak pressure $F_{\rm ya}^{\max }$ of the radial compression physical test of cotton stalks is taken as the target value, and the fitted equations are solved numerically to derive the optimal combinations of the significance parameters.

#### Verification test

To verify the accuracy of the calibration results of the parameters of the cotton stalks and the shear performance of the simulation model, a discrete element model of cotton stalks with a diameter of 7.5 mm and a length of 150 mm is constructed using the same method as that in the simulated compression test on cotton stalk. The simulated bending and shear tests on cotton stalks are carried out according to the optimal combination of parameters derived from the *BBD* test, and then the simulation results are compared with the results of the physical test.

## Results and discussion

### Analysis of the results of cotton stalk physical tests

Based on the results of the tensile, radial compression, three-point bending, and shear tests of cotton stalks, the mechanical properties of cotton stalks are obtained, as shown in Table 2. The $F_{\rm la}^{\max }$, $F_{\rm ya}^{\max }$, $F_{\rm Wa}^{\max }$, and $F_{\rm ja}^{\max }$ are 814.2 N, 244.7 N, 97.6 N, and 221.8 N, respectively. Then, the $\sigma _{\rm l}^{\max }$, $\sigma _{\rm y}^{\max }$, $\sigma _{\rm w}^{\max }$, and $\sigma _{\rm j}^{\max }$, can be determined with Eqs. (1)–(4). The standard deviation of the test results is attributed to the individual differences between the samples.

**Table 2:** Physical test results of cotton stalk.

Stalk properties	Min	Max	Average	Standard deviation
Diameter(mm)	6.8	8.27.49	0.21	
$F_{\rm la}^{\max }$ (N)	711.3	941.5	814.17	88.2
$F_{\rm ya}^{\max }$(N)	191.5	305.2	244.70	40.24
$F_{\rm wa}^{\max }$ (N)	105.5	144.6	120.40	12.60
$F_{\rm ja}^{\max }$(N)	174.8	287.5	221.8	41.59
$\sigma _l^{\max }$(Pa)	1.61 × 10$^7$	2.13 × 10$^7$	1.84 × 10$^7$	3.59 × 10$^5$
$\sigma _y^{\max }$(Pa)	1.70 × 10$^6$	2.71 × 10$^6$	2.24 × 10$^6$	1.69 × 10$^3$
$\sigma _w^{\max }$ (Pa)	5.73 × 10$^4$	7.86 × 10$^4$	6.59 × 10$^4$	4.12 × 10$^2$
$\sigma _j^{\max }$(Pa)	7.91 × 10$^6$	1.30 × 10$^7$	1.0 × 10$^7$	5.63 × 10$^3$

**Figure 5:**
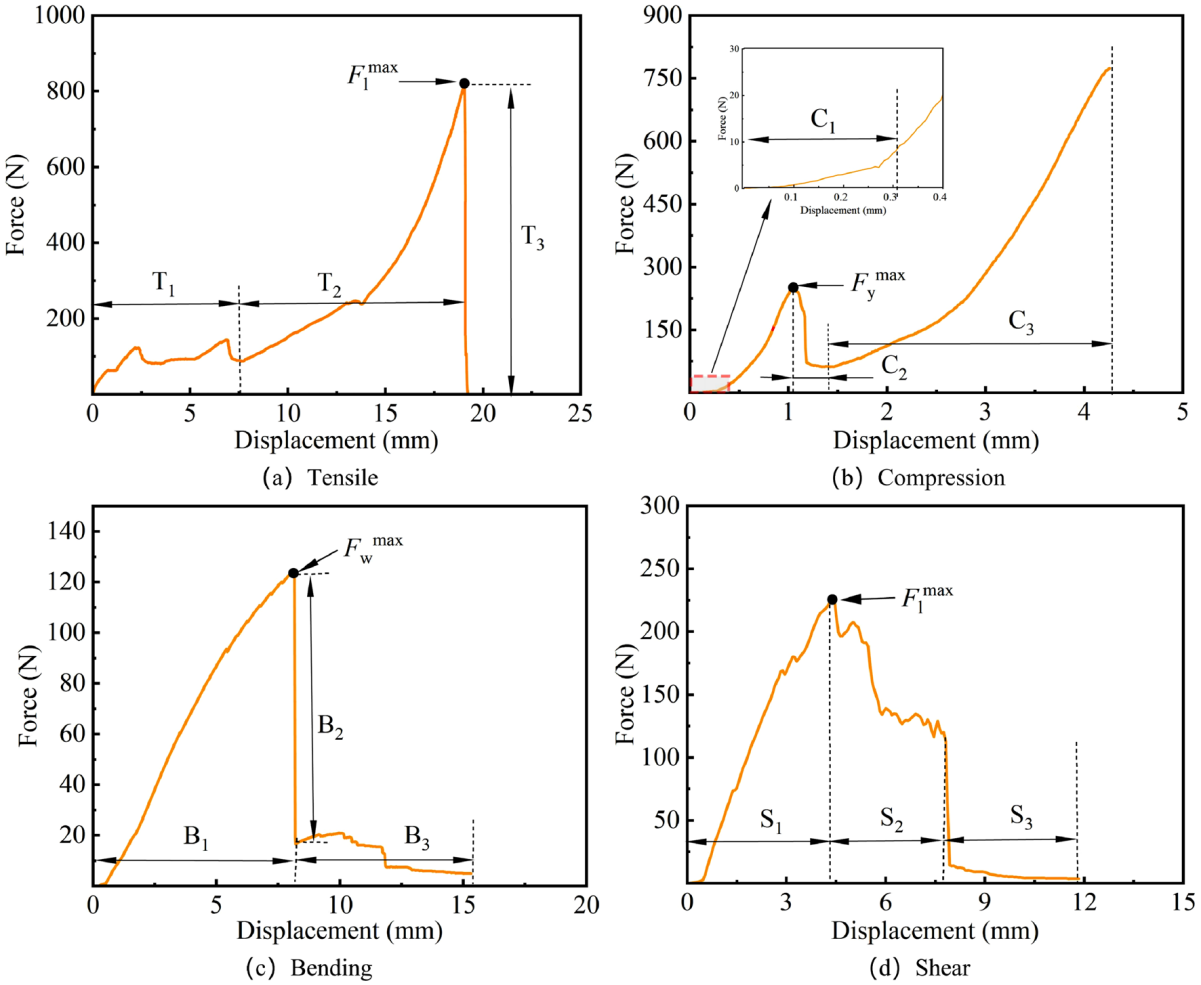
*F*–*x* curves of cotton stalk obtained from the tensile, compression bending, and shear tests.

Figure 5a indicates the *F*–*x* curve of the tensile test of cotton stalk, and the evolution of the *F*–*x* curve can be divided into the initial fluctuation stage $T_{\rm 1}$, the linear growth stage $T_{\rm 2}$, and the sudden drop stage $T_{\rm 3}$. In $T_{\rm 1}$, the tensile force fluctuates with the rise of the fixture, which is due to the small cracks of the cotton stalks extruded by the upper and lower fixtures and the slight sliding caused by the upper and lower ends of the fixtures. As the fixture continues to rise, the cotton stalks are fixed relative to the fixture, and the tensile force experiences a linear growth trend. With the further rise of the fixture, when the tensile force is equal to the peak value of the tensile force $F_{\rm l}^{\max }$, the cotton stalks brittlely break, the tensile force is significantly reduced, and the test is terminated.

Figure 5b shows the *F*–*x* curve of the ring compression test of cotton stalks, and the evolution of the *F*–*x* curve could be divided into the slow-rising stage $C_{\rm 1}$, the linear growth stage $C_{\rm 2}$, the sudden drop stage $C_{\rm 3}$, and the rising stage $C_{\rm 4}$. In $C_{\rm 1}$, the pressure exhibits a slow rising trend, which may be related to the simplification of the model or the skin of cotton stalks. The force shows a linear growth trend with the continuous drop of the upper plate, indicating that the cotton stalks gradually become denser under the load and exhibit compressive properties. When the applied load is greater than the $F_{\rm y}^{\max }$, the cotton stalks break and the force decreases significantly. Subsequently, as the cotton stalks are flattened and recover their ability to resist deformation, the force shows an increasing trend again and the test is terminated.

The *F*–*x* curve of the bending test of cotton stalk is illustrated in Fig. 5c, and the evolution of the *F*–*x* curve can be divided into the linearly increasing stage $B_{\rm 1}$, the sudden drop stage $B_{\rm 2}$, and the fluctuating stage $B_{\rm 3}$. In $B_{\rm 1}$, the bending force shows a linear increasing trend, indicating that the cotton stalks yield bending deformation under the action of the loads and exhibit bending resistance. With the continuous drop of the upper-pressure tube, when the applied load is greater than the $F_{\rm W}^{\max }$ of cotton stalks, the bottom end of the middle of the cotton stalks begins to break, and the bending force significantly reduces. The bending force tends to fluctuate up and down and the test is terminated.

Figure 5d shows the *F*–*x* curve of the shear test of cotton stalk, and the evolution of the *F*–*x* curve can be divided into the linear rise stage $S_{\rm 1}$, the fluctuation decline stage $S_{\rm 2}$, and the sudden drop stage $S_{\rm 3}$. The shear force of the cotton stalk is related to the shear cross-sectional area. With the downward feeding of the movable knife, the shear cross-sectional area of the cotton stalk gradually increases, and the shear force shows a linear increase trend. When the movable knife feeds to the position of 3.5-4 mm, i.e., at the radius distance of the cotton stalk, the shear force reaches the $F_{\rm j}^{\max }$. With the gradual decrease of the shear cross-sectional area, the mechanical state of the cotton stalks pith core is not stable, and the shear force shows a gradually fluctuating downward trend. The shear force shows a gradual and rapid decline trend as the movable knife continues to descend, after which the cotton stalks are sheared and the test is terminated.

### Design and parameters of simulation tests

#### Analysis of ${\textbf {PBD}}$test results

Given the large number of microscopic parameters of cotton stalks and the particles in the flat-joint model, as well as the large computer load during the simulation process, the *PBD* test method is used to design the test scheme through the Design-Expert software with the $F_{\rm y}^{\max }$ in the compression test of cotton stalks as the response value, and with the 13 parameters in Table 1 as the test factors. The design and the results of the test scheme are shown in Table 3.

**Table 3:** Design and results of *PBD* test.

No.	Factors													$F_{\rm y}^{max}$(N)
	$X_{\rm 1}$	$X_{\rm 2}$	$X_{\rm 3}$	$X_{\rm 4}$	$X_{\rm 5}$	$X_{\rm 6}$	$X_{\rm 7}$	$X_{\rm 8}$	$X_{\rm 9}$	$X_{\rm 10}$	$X_{\rm 11}$	$X_{\rm 12}$	$X_{\rm 13}$	
1	1	− 1	1	1	1	1	1	− 1	1	− 1	1	− 1	− 1	332.4
2	1	− 1	1	1	− 1	− 1	1	1	1	1	− 1	1	− 1	94.6
3	1	1	− 1	1	− 1	1	− 1	− 1	− 1	− 1	1	1	− 1	114.7
4	− 1	1	− 1	1	− 1	− 1	− 1	− 1	1	1	− 1	1	1	97.3
5	1	− 1	1	− 1	1	− 1	− 1	− 1	− 1	− 1	1	− 1	1	98.1
6	− 1 -	1	− 1	− 1	− 1	− 1	− 1	− 1	− 1	− 1	− 1	− 1	− 1	84.3
7	− 1	1	1	− 1	− 1	1	1	1	1	− 1	1	− 1	1	167.4
8	1	1	− 1	− 1	− 1	1	1	− 1	1	1	− 1	− 1	1	129.6
9	1	1	− 1	1	1	− 1	1	1	1	1	1	− 1	1	223.4$*$
10	1	1	1	− 1	− 1	− 1	− 1	1	1	− 1	1	1	− 1	89.1
11	1	1	1	1	− 1	1	− 1	1	− 1	− 1	− 1	− 1	1	177.2$*$
12	− 1	− 1	1	1	− 1	1	1	− 1	− 1	1	1	1	1	160.4
13	− 1	1	1	1	1	− 1	1	− 1	1	− 1	− 1	− 1	− 1	214.7$*$
14	− 1	− 1	− 1	− 1	1	1	− 1	1	1	− 1	− 1	1	1	180.9$*$
15	1	1	− 1	− 1	1	1	1	1	− 1	1	− 1	1	− 1	170.4
16	− 1	1	− 1	− 1	− 1	− 1	1	1	− 1	1	1	− 1	− 1	101.7
17	1	1	1	− 1	1	− 1	1	− 1	− 1	− 1	− 1	1	1	112.9
18	− 1	− 1	− 1	1	1	− 1	1	1	− 1	− 1	1	1	1	225.8$*$
19	− 1	1	1	− 1	1	1	− 1	− 1	1	1	1	1	− 1	136.2
20	− 1	− 1	1	1	1	1	− 1	1	− 1	1	− 1	− 1	− 1	310.23

Note:$*$ indicates that the value is within the interval of the physical test result.

As indicated in Table 3, the $F_{\rm y}^{\max }$ for the 20 simulation tests varies from 89.1 to 332.4 N. For the range of $F_{\rm y}^{\max }$ in the physical compression tests, tests numbered 9, 13 and 18 are found to meet the requirements, and $F_{\rm y}^{\max }$ values are 223.4 N, 214.7 Nand 225.8 N.

The results of the tests in Table 3 are analyzed by ANOVA, and the results are shown in Table 4.

**Table 4:** ANOVA of the *PBD* test.

Source	Sum of Squares	df	Mean Square	F-value	*p* value
Model	90440.02	13	6956.92	6.94	0.0129*
$X_\mathrm{{1}}$:$K_{n\mathrm b}$/(N/m)	931.61	1	931.61	0.9295	0.3722
$X_\mathrm{{2}}$:$K_{\rm w}$(N/m)	1795.51	1	1795.51	1.79	0.2292
$X_\mathrm{{2}}$:$k_{\rm g}$	497	1	497	0.4959	0.5077
$X_\mathrm{{4}}$:*E*(x-x)/(Pa)	23,126.8	1	23,126.8	23.08	0.003**
$X_\mathrm{{5}}$:$\sigma _n$(x-x)/(Pa)	31,102.38	1	31,102.38	31.03	0.0014**
$X_\mathrm{{6}}$:$\sigma _s$(x-x)/(Pa)	14,445.31	1	14,445.31	14.41	0.009**
$X_\mathrm{{7}}$:*E*(p-p)/(Pa)	1970.11	1	1970.11	1.97	0.2105
$X_\mathrm{{8}}$:$\sigma _n$(p-p)/(Pa)	3382.6	1	3382.6	3.38	0.1158
$X_\mathrm{{9}}$:$\sigma _s$(p-p)/(Pa)	603.9	1	603.9	0.6025	0.4671
$X_\mathrm{{10}}$:*E*(x-p)/(Pa)	1575.31	1	1575.31	1.57	0.2566
$X_\mathrm{{11}}$:$\sigma _n$(x-p)/(Pa)	297.22	1	297.22	0.2966	0.6057
$X_\mathrm{{12}}$:$\sigma _s$(x-p)/(Pa)	10,428.74	1	10,428.74	10.41	0.018*
$X_\mathrm{{13}}$:$\mu _c$	283.5	1	283.5	0.2829	0.6139
Pure Error	6013.46	6	1002.24	6.94	0.0129*
Cor Total	96,453.49	19	6956.92	0.9295	0.3722

*Indicates that the value is significant, p < 0.05; ** indicates that the value is highly significant, *p* < 0.01.

It is observed from Table 4 that the P value of the model is 0.0129 (<0.05), and the R$^{2}$ coefficient of the model is 93.8 (>93), which indicates that the models can explain more than 93 of the changes in the response value and that the predicted values have a high correlation with the actual values, with the small errors. It indicates that the regression equation fitted by the model is consistent with the actual situation and can reflect the degree of influence of factors $X_{\rm 1}-X_{\rm 13}$ on the response value of the pressure. The regression model describing the relationship between the the $F_{\rm y}{\max }$ and various factors is obtained as follows:

6 $$\begin{aligned} \begin{aligned} F_{\rm y}{\max }&=161.06-6.83X_{\rm 1}-9.47X_{\rm 2}-4.98X_{\rm 3}+34.0X_{\rm 4}+39.44X_{\rm 5}+26.88X_{\rm 6}+9.92X_{\rm 7}\\&\quad +13.01X_{\rm 8}+5.5X_{\rm 9}-8.88X_{\rm 10}+3.85X_{\rm 11}-22.84X_{\rm 12} -3.76X_{\rm 13} \end{aligned} \end{aligned}$$

According to Eq. (6), the primary term coefficients are obtained for each factor on $F_{\rm y}^{\max }$ in the order of predominance of $X_{\rm 5}$, $X_{\rm 4}$, $X_{\rm 6}$, $X_{\rm 12}$, $X_{\rm 8}$, $X_{\rm 7}$, $X_{\rm 2}$, $X_{\rm 10}$, $X_{\rm 9}$, $X_{\rm 13}$, $X_{\rm 11}$, $X_{\rm 13}$. Analyzing the *p* values of the factors, it is observed that the factors $X_{\rm 4}$, $X_{\rm 5}$, $X_{\rm 6}$, and $X_{\rm 12}$ are significant, and the others are not significant.

#### Analysis of the steepest climb test results

The degree of influence and significance of the 13 factors on the $F_{\rm y}{\max }$ are analyzed by the *PBD* test. However, it is not clear whether each factor affects the change rule of the *F*–*x* curve. To further narrow down the range of significant factors and explore the influence of each factor on the trend of the *F*–*x* curve, the steepest climb test is carried out for the factors $X_{\rm 4}$, $X_{\rm 5}$, $X_{\rm 6}$, $X_{\rm 12}$, while the rest of the factor levels are selected as intermediate values. The $F_{\rm y}^{\max }$ and the $\delta$ are taken as response values. The test scheme and the results are shown in Table 5.

**Table 5:** Design and results of steepest ascent test.

No.	Factors				$F_{\rm y}^{\max }$(N)	$\delta$ (%)
	$X_{\rm 4}$	$X_{\rm 5}$	$X_{\rm 6}$	$X_{\rm 12}$		
1	5.0 × 10$^7$	1.0 × 10$^6$	5.0 × 10$^6$	1.0 × 10$^7$	63.9	73.90
2	6.25 × 10$^7$	1.25 × 10$^6$	6.25 × 10$^6$	1.25 × 10$^7$	77.2	68.50
3	7.5 × 10$^7$	2.5 × 10$^6$	7.5 × 10$^6$	2.5 × 10$^7$	128.8	47.40
4	8.75 × 10$^7$	3.75 × 10$^6$	8.75 × 10$^6$	3.75 × 10$^7$	171.2	30.00
5	1.0 × 10$^8$	5.0 × 10$^6$	1.0 × 10$^7$	5.0 × 10$^7$	214.75	12.20
6	1.25 × 10$^8$	6.25 × 10$^6$	1.25 × 10$^7$	6.25 × 10$^7$	262.8	7.40
7	2.5 × 10$^8$	7.5 × 10$^6$	2.5 × 10$^7$	7.5 × 10$^7$	335.6	37.10
8	3.75 × 10$^8$	8.75 × 10$^6$	3.75 × 10$^7$	8.75 × 10$^7$	398.8	62.90
9	5.0 × 10$^8$	1.0 × 10$^7$	5.0 × 10$^8$	1.0 × 10$^8$	461.1	88.40

The rest of the factors are the median values used.

Table 5 shows the design scheme and results of the steepest climb test, it demonstrates that as $X_{\rm 4}$, $X_{\rm 5}$, $X_{\rm 6}$, and $X_{\rm 12}$ gradually increase, the $F_{\rm y}^{\max }$ of cotton stalks also increases, and the $\delta$ shows a tendency to decrease and then increase. The $\delta$ is the smallest in the 6th group of tests, with a value of 7.40%. The maximum $\delta$ of the 9th group of tests is 88.40%. The results of the 9 tests are significantly different.

**Figure 6:**
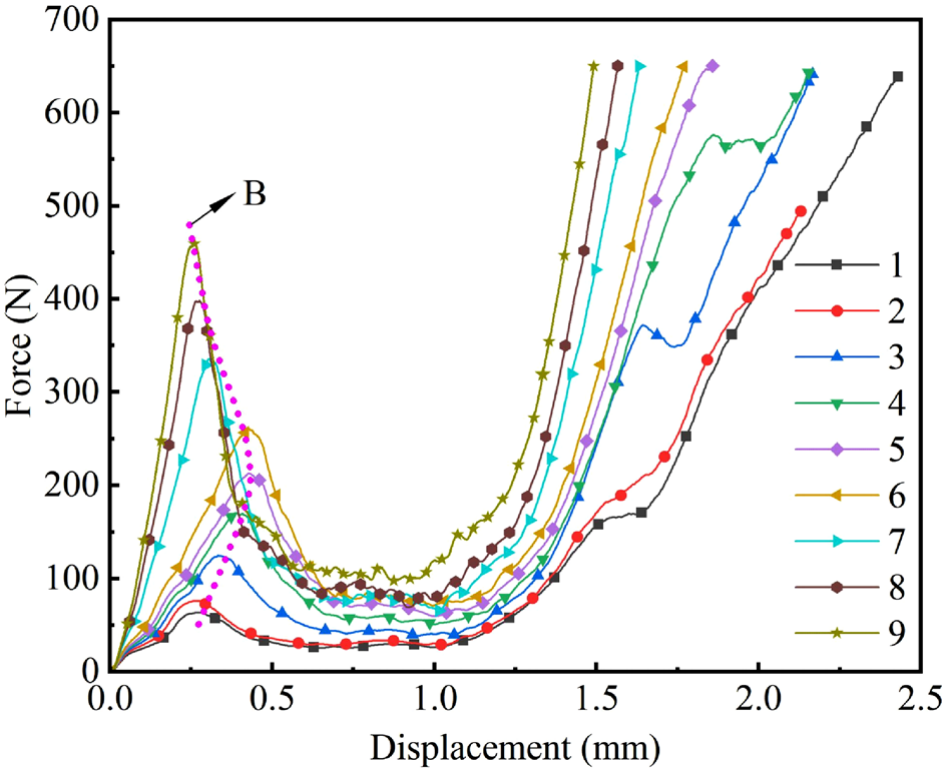
Effects of different parameters on $F_{\rm y}^{\max }$ in *PBD* test.

The *F*–*x* curves are plotted for the different test schemes in Table 5, as shown in Fig. 6. The results show that the $F_{\rm y}^{\max }$ increased with the increase of the value of the test factor, which was consistent with the analysis of the results of the *PBD* test. From the change rule of the *F*–*x* curve and the change of the compression state of the cotton stalk model, it is observed that the whole compression process is mainly divided into the linear growth stage, the sudden drop stage, and the rising stage. For the linear growth stage: the force grows approximately linearly and finally reaches the $F_{\rm y}^{\max }$, indicating that the particles in the model are internally stressed due to mutual contact. For the sudden drop stage: as the upper-pressure plate, cotton stalks xylem and pith core continue to fall, the internal stress in the model exceeds the ultimate strength in turn, leading to collapse at the weak position. The force shows a rapid decline trend. For the rising stage: with the upper pressure plate falling further, the internal stress in the model is greater than the ultimate strength. The particles in the model aggregate and the internal organization of the model recovers the deformation resistance. The force shows a linear rising trend, and the test is terminated.

For the nine *F*–*x* curves, the curves in the rising phase again are steeper with the increasing test factor level, but the wave peaks show a gradual backward and then forward trend, as shown in the fitted line *B*. This indicates that the *F*–*x* curves are related to the parameter variations of the cohesive modulus *E*, normal cohesive strength $\sigma _{\rm n}$, and tangential cohesive strength $\sigma _{\rm S}$. The results confirm that $X_{\rm 4}$, $X_{\rm 5}$, $X_{\rm 6}$, and $X_{\rm 12}$ not only affect the $F_{\rm y}^{\max }$ but also have a significant effect on the direction of the *F*–*x* curve. Based on the curve direction and physical test measurement range, the *BBD* test is conducted with the steepest climb test parameter of group 6 as the center value, and the test parameters of groups 5 and 7 as the low-level and high-level values, respectively.

#### Analysis of BBD test results

To obtain the optimal combination of simulation parameters for the cotton stalks model, the test factors and levels derived from the steepest climb test are used. The results are fitted and analyzed to develop a second-order regression model describing the relationship between the $F_{\rm y}^{\max }$ and the influencing factors $X_{\rm 4}$, $X_{\rm 5}$, $X_{\rm 6}$, and $X_{\rm 12}$ with a quadratic polynomial equation shown in Eq. (7). The regression model is analyzed and the test results are shown in Table 6 Design and results of *BBD* test.

7 $$\begin{aligned} \begin{aligned} F_{\rm y}{\max }&=238.44+5.49X_{\rm 4}+14.30X_{\rm 5}+3.59X_{\rm 6}+2.88X_{\rm 12}-0.80X_{\rm 4}X_{\rm 5}+0.325X_{\rm 4}X_{\rm 6}-0.85X_{\rm 4}X_{\rm 12}\\&\quad +3.28X_{\rm 6}X_{\rm 6}+0.875X_{\rm 5}X_{\rm 12}-1.63X_{\rm 6}X_{\rm 12}+0.6175X_{\rm 6}X_{\rm 4}^2-0.27X_{\rm 5}^2-1.18X_{\rm 6}^2+0.805X_{\rm 12}^2 \end{aligned} \end{aligned}$$

**Table 6:** Design and results of *BBD* test.

No.	Factors				$F_{\rm y}^{\max }$(N)	$\delta$ (%)
	$X_{\rm 4}$	$X_{\rm 5}$	$X_{\rm 6}$	$X_{\rm 12}$		
1	0	0	1	− 1	237.1	3.11
2	− 1	0	0	− 1	231.1	5.56
3	0	0	− 1	− 1	230.2	5.93
4	1	0	0	1	247.4	1.10
5	− 1	0	− 1	0	227.3	7.11
6	0	1	− 1	0	244.3	0.16
7	0	− 1	− 1	0	223.9	8.50
8	0	0	− 1	1	239.8	2.00
9	1	0	0	− 1	244.3	0.16
10	− 1	− 1	0	0	215.2	12.06
11	0	1	0	1	254.6	4.05
12	0	0	0	0	238.5	2.53
13	0	0	0	0	238.4	2.57
14	0	− 1	0	1	231.5	5.39
15	1	0	− 1	0	238.8	2.41
16	1	0	1	0	251.1	2.62
17	0	1	0	− 1	250.2	2.25
18	1	1	0	0	258.3	5.58
19	− 1	1	0	0	250.8	2.49
20	0	− 1	0	− 1	223.6	8.62
21	0	0	0	0	238.4	2.57
22	0	0	1	1	240.2	1.84
23	0	− 1	1	0	223.6	8.62
24	− 1	0	1	0	238.3	2.62
25	0	0	0	0	238.5	2.53
26	0	0	0	0	238.4	2.57
27	− 1	0	0	1	237.6	2.90
28	0	1	1	0	257.1	5.06
29	1	− 1	0	0	225.9	7.68

As demonstrated in Table 6, the $F_{\rm y}^{\max }$ in the 29 simulation tests ranges from 215.2 to 258.3 N, and the $\delta$ varies from 0.16 to 12.06%. The $F_{\rm y}^{\max }$ values in 29 simulation tests match well with those in the physical compression test, which are all in line with the test requirements. For the $F_{\rm ya}^{\max }$, the minimum and maximum values of $\delta$ are 0.16% and 12.06%, respectively, indicating that the test factors and levels used in this test are close to the optimal combination of simulation parameters of cotton stalks.

**Table 7:** ANOVA of *BBD* test.

Source	Sum of Squares	df	Mean Square	F-value	*p* value
Model	3148.37	14	224.88	29.55	<0.0001**
$X_{\rm 4}$	357.52	1	357.52	46.98	<0.0001**
$X_{\rm 5}$	2453.88	1	2453.88	322.43	<0.0001**
$X_{\rm 6}$	154.8	1	154.8	20.34	0.0005**
$X_{\rm 12}$	99.76	1	99.76	13.11	0.0028**
$X_{\rm 4}X_{\rm 5}$	2.56	1	2.56	0.3364	0.5711
$X_{\rm 4}X_{\rm 6}$	0.4225	1	0.4225	0.0555	0.8171
$X_{\rm 4}X_{\rm 12}$	2.89	1	2.89	0.3797	0.5476
$X_{\rm 5}X_{\rm 6}$	42.9	1	42.9	5.64	0.0324*
$X_{\rm 5}X_{\rm 12}$	3.06	1	3.06	0.4024	0.5361
$X_{\rm 6}X_{\rm 12}$	10.56	1	10.56	1.39	0.2584
$X_{\rm 4}^2$	2.47	1	2.47	0.325	0.5777
$X_{\rm 5}^2$	0.4729	1	0.4729	0.0621	0.8068
$X_{\rm 6}^2$	9.07	1	9.07	1.19	0.2934
$X_{\rm 12}^2$	4.2	1	4.2	0.5523	0.4697
Residual	106.55	14	7.61		
Cor Total	3254.92	28	10.65		

*Indicates that the value is significant, $p<0.05$; ** indicates that the value is highly significant, $p<0.01$.

**Figure 7:**
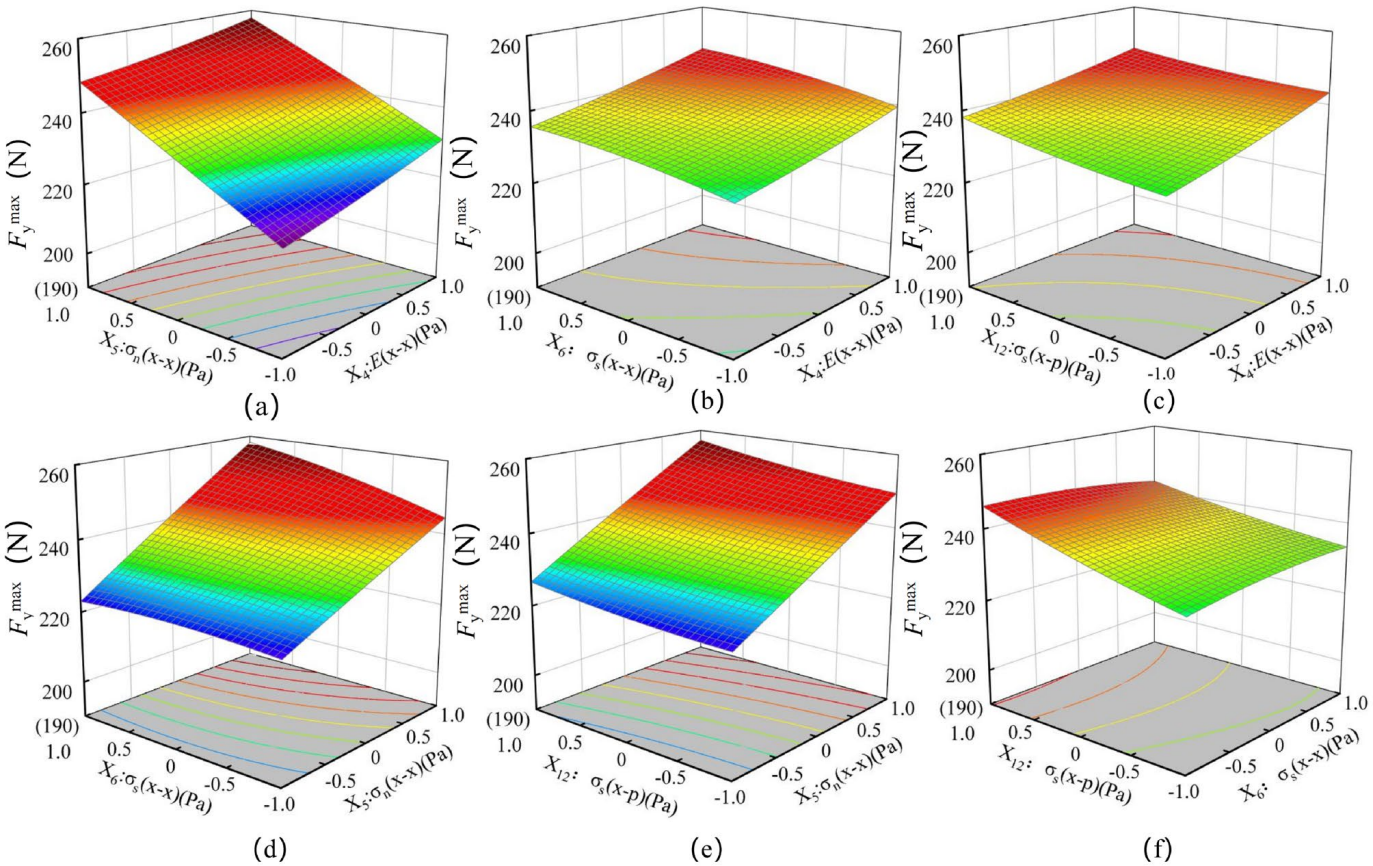
Influence of the interaction term on $F_{\rm y}^{\max }$: (**a**) Influence of $X_{\rm 4}$ and $X_{\rm 5}$ on $F_{\rm ya}^{\max }$; (**b**) Influence of $X_{\rm 4}$ and $X_{\rm 6}$ on $F_{\rm ya}^{\max }$; (**c**) Influence of $X_{\rm 4}$ and $X_{\rm 12}$ on $F_{\rm ya}^{\max }$; (**d**) Influence of $X_{\rm 5}$ and $X_{\rm 6}$ on $F_{\rm ya}^{\max }$; (**e**) Influence of $X_{\rm 5}$ and $X_{\rm 12}$ on $F_{\rm ya}^{\max }$; (**f**) Influence of $X_{\rm 6}$ and $X_{\rm 12}$ on $F_{\rm ya}^{\max }$.

The test results in Table 6 are analyzed by variance analysis, and the results are presented in Table 7. The results confirm that the *p* value of the equation model is less than 0.0001, which presents extreme significance ($p<$0.01) and is statistically significant, indicating that the $F_{\rm y}$ and the $\delta$ with the test factors present extreme significance. The coefficient of determination $R^{\rm 2}$=0.9673 and the corrected coefficient of determination $R_{\rm adj}$= 0.9345 indicate that the quadratic regression equation fitted by the model is consistent with the actual situation. The predictive coefficient $R_{\rm pre}$=0.9090 and the corrected coefficient of determination $R_{\rm adj}$=0.9345 are the same, with a difference of less than 0.3, which demonstrates a high reliability. The results in Table 7 show that the primary terms $X_{\rm 4}$, $X_{\rm 5}$, $X_{\rm 6}$, and $X_{\rm 12}$ are highly significant, and the order in which each factor affects force is: $X_{\rm 5}>X_{\rm 4}>X_{\rm 6}>X_{\rm 12}$. The significance of the secondary terms is not significant. Only the $X_{\rm 5}$,$X_{\rm 6}$ interaction term has a significant effect on the $F_{\rm y}$, and the rest of the interaction terms have insignificant effects. The response surface plots of the interaction effect of each factor on the force are obtained according to the regression equation, as shown in Fig. 7.

Taking the $F_{\rm ya}^{\max }$ of 244.7 N of the cotton stalks in the physical ring compression test as the target value, the simulation parameters are optimized. The fitted equations are solved numerically with the Design-Expert software to obtain the optimal combinations of the significance parameters. The *E*(x-x), $\sigma _{\rm n}$(x-x), $\sigma _{\rm s}$(x-x), and $\sigma _{\rm s}$(x-p) are 0.85$\times$10$^8$Pa, 6.0525$\times$10$^6$ Pa, 2.11$\times$10$^7$Pa, and 1.31$\times$10$^7$Pa, respectively, and the rest of the factor levels are selected as intermediate values. The above optimal parameter combinations are adopted for the simulation tests. The simulated $F_{\rm y}^{\max }$ is 244.35 N, and the relative error to the $F_{\rm y}^{\max }$ of the physical test is 0.14%, which indicates that the results of the simulated and physical tests are the same. The results of the simulated and physical tests are in good agreement, indicating that the optimal parameter combinations derived from the simulation test are accurate and reliable, and can be used for discrete element simulation of the ring compression test of cotton stalks.

### Verification of the test

#### Verification of ring compression test

**Figure 8:**
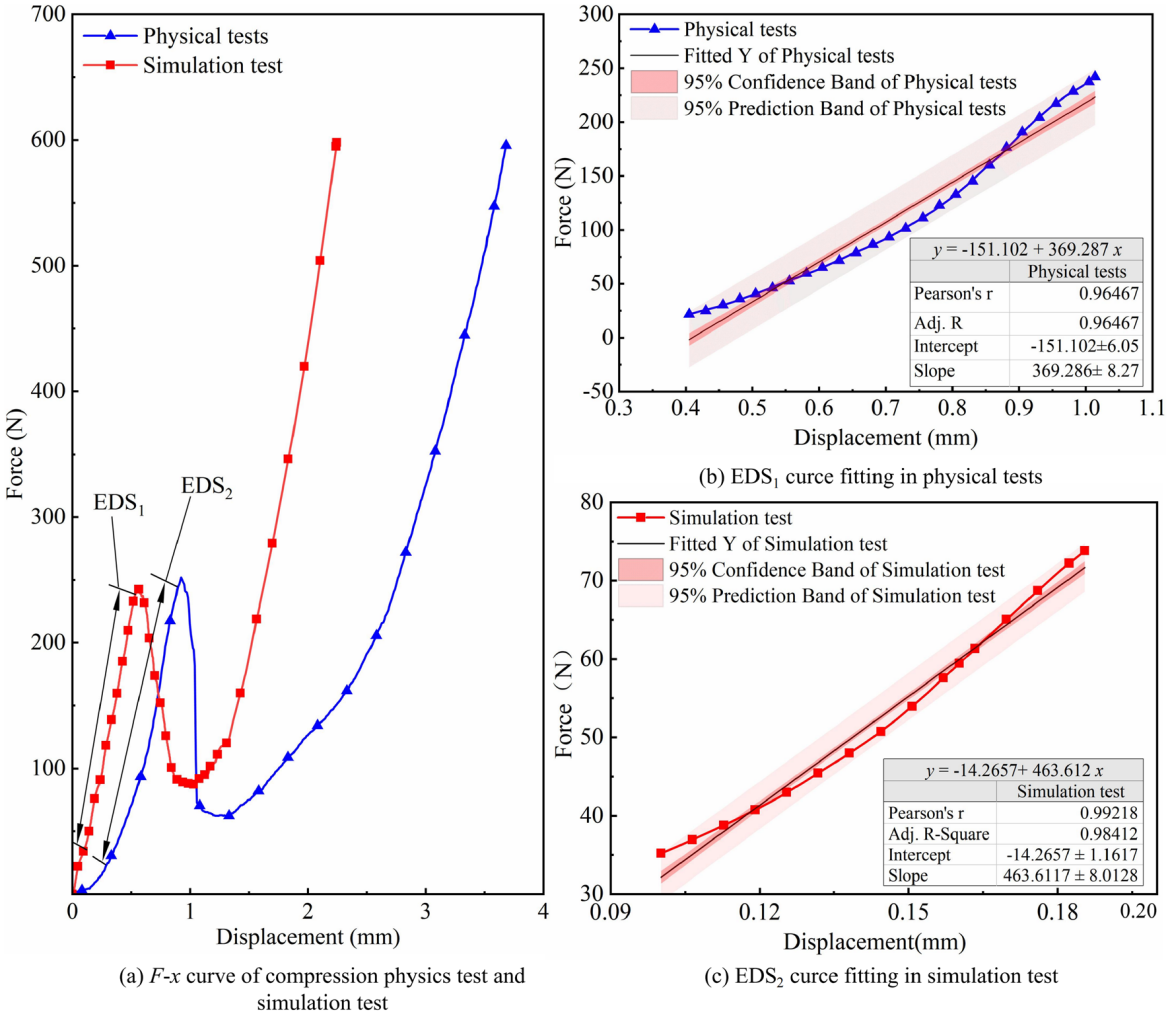
*F*–*x* curve and elastic deformation stage of simulated and physical compression tests.

Compression validation tests are carried out using PFC6.0$^{\rm 3D}$ for the optimal combination of simulation parameters in the cotton stalk model, and *F*–*x* curves are extracted, as shown in Fig. 8. Comparison of the *F*–*x* curves in the simulated and physical tests shows that the peak forces of the cotton stalks obtained from the two tests are similar and that the trend of the *F*–*x* curves of the two tests is the same. It is demonstrated that the established model of the cotton stalks can reflect the change rule of the force with the displacement in the compression process. The different limiting positions of the physical and simulated compressive strengths of the cotton stalks may be attributed to the large difference in the diameter and wall thickness of the cotton stalk specimens, which provide different deformation positions of the limiting pressure. In addition, during the initial compression stage, the simulated test values all show a linear increasing trend, while the physical test values show a slowly increasing trend in the 0–0.3 mm displacement section, followed by a linear increasing trend. The reason for this situation may be related to the simplified treatment or the water content of the cotton stalks^17^, which is in line with the findings of Zhao et al.^31^.

The linear rise stage in the *F*–*x* curves is the main stage for analyzing the mechanical property parameters of cotton stalks. The *F*–*x* curves of the linear rise stage in the two tests are extracted. The curves of the linear rise stage EDS$_1$ (0.4–1.0 mm) in the physical test are shown in Fig. 8b, and the curves of the linear rise stage EDS$_2$ (0.1–0.6 mm) in the simulated test are shown in Fig. 8c. The curves are fitted to the *F*–*x* curves of the two tests using the Origin software to obtain the fitted equations of the curves, with higher values of R$^2$ and R$_{\rm adj}$ indicating better-fitted equations. In the fitted equations, R$^2$ and R$_{\rm adj}$ in the simulated test are 0.98441 and 0.98412, respectively, while those in the physical test are 0.96515 and 0.96467, respectively, both of which are greater than 0.96, which indicates that the two fitted equations are extremely accurate. The slopes in the simulated and physical tests are $463.61\pm 8.01$ and $369.29\pm 8.27$, respectively, with a relative error of 2.03%. This indicates that the force and deformation of cotton stalks in the elastic phase of the simulated test are highly consistent with those of the physical test.

**Figure 9:**
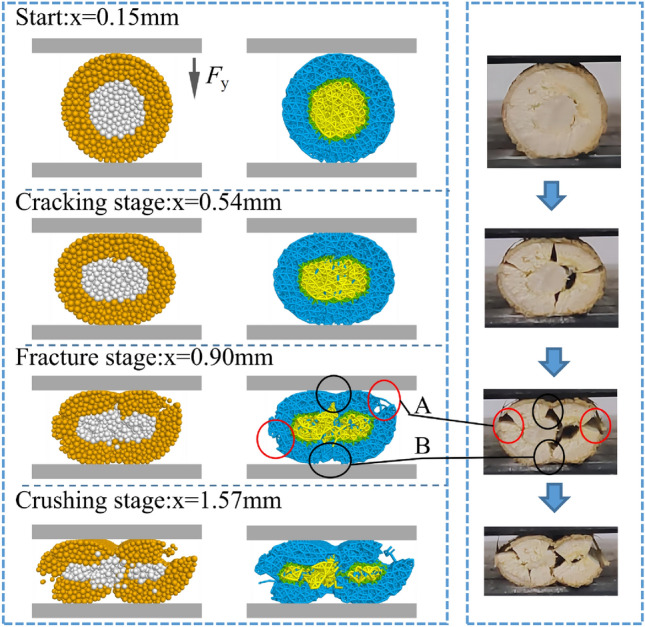
The compressive deformation process of the cotton stalk in physical and simulation tests.

The compressive deformation process of cotton stalks is shown in Fig. 9. The simulated and physical compression deformation processes of cotton stalks are shown in Fig. 9a and b, respectively. By observing the changes in the cross-section of the cotton stalks, the compression processes of the cotton stalks all show three stages: the emergence of the crack stage, the fracturing stage, and the crushing stage. In the emergence of the crack stage, with the feeding of the upper platen, the load on the cotton stalk increases, the cumulative shape variable of the cross-section increases with rapidly changing from circular to elliptical, and small cracks appear. In the crushing stage, openings are formed on the left and right sides as well as the upper and lower sides of the cotton stalks, and longitudinal and transversal cracks occur, as shown at positions A and B in Fig. 9. As the upper platen continues to feed, the cracks continue to expand, with the upper longitudinal and transversal cracks passing through, as shown in Fig. 9b.

With the continuous feeding of the upper platen, the cracks keep expanding with the upper longitudinal and transversal cracks passing through, and the cotton stalks form a 4-flap structure. Cotton stalks reach the stage of compression collapse, and the cotton stalks are continuously compressed, and the upper and lower layers of the cotton stalks are compacted and overlapped. The compression force shows a linear growth trend, and the compression test terminates.

**Figure 10:**
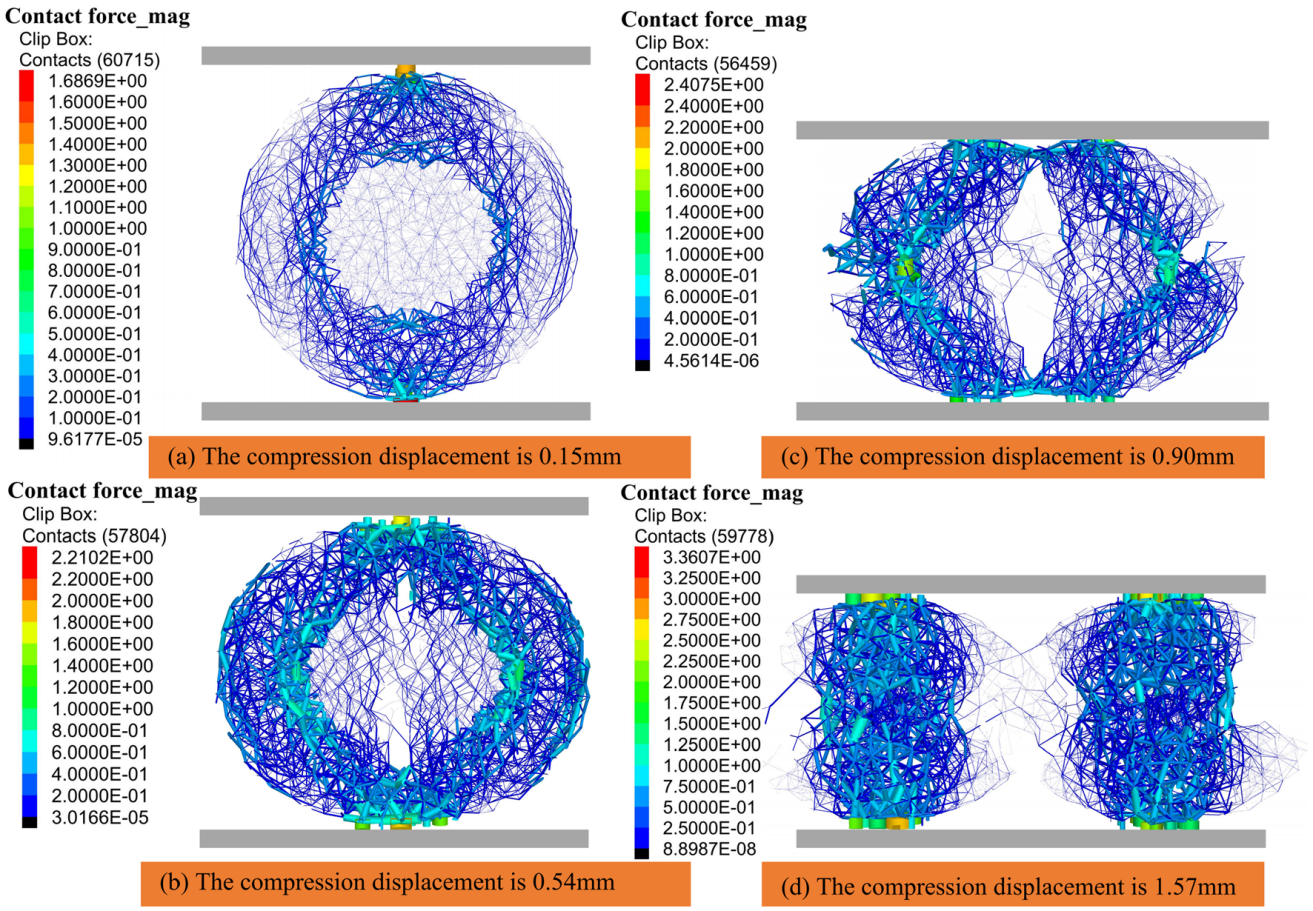
The contact force chain of the cotton stalk in the compression process.

Figure 10 presents a chain diagram of the contact force during compression, where the thickness and color of the line indicate the magnitude of the stress, the thickness of the line indicates the magnitude of the stress^17^. As shown in Fig.10a, when the compression displacement is 0.15 mm, the number of contact force chains is 60,715. The contact force at the upper and lower compression plates begins to increase, and the maximum contact force is 0.5 N. The compressive stresses are mainly centered at the left and right ends of the inner ring of the upper and lower compression plates and the inner rings of the wood section. The tensile stresses begin to appear at the left and right sides of the model and the upper and lower ends of the inner ring of the wood section. The contact forces in the pith part are at a small level. As shown in Fig. 10b, when the compression displacement is 0.54 mm, the contact force, tensile stress, and compressive stress keep increasing, and the number of contact force chains is 57,804. The tensile stress chains at the left and right sides of the model are broken, and the force chains in the pith part begin to break from top to bottom, which is consistent with the phenomenon of the physical test. As indicated in Fig. 10c, when the compression displacement is 0.90 mm, the number of contact force chains is 56,495, and the number of contact force chains decreases, which indicates that the cotton stalks are crushed. The force chains on both sides of the stalks as well as those in the middle of the medullary core increase gradually, which is mainly manifested as compressive stress. As shown in Fig. 10d, when the compression displacement is 1.57 mm, the number of contact force chains is 59,778. There is only a small number of force chains in the middle part of the model, which indicates that the middle and the two sides of the cotton stalks are broken. The two sides of the model are also completely disconnected from the force chains, which are mainly manifested as compressive stresses that are concentrated in the compaction zone of the model. This is consistent with the phenomenon of the compression collapse stage in the physical test.

The analysis of the compression process of cotton stalks demonstrates that the compression process and crushing characteristics of cotton stalks in the simulated and physical tests are the same. The established discrete element model of cotton stalks can accurately characterize the mechanical properties of cotton stalks under ring compression, which can be used for the model establishment of cotton stalks and the optimization of parameters.

#### Verification of three-point bending test

To verify the accuracy of the calibration results of the microscopic parameters of the cotton stalks, a model of cotton stalks with a diameter of 7.50 mm and a length of 150.0 mm is constructed using the same method as that in the simulated compression test, and the optimal parameter combinations obtained from the compression test are adopted for the simulated three-point bending test.

**Figure 11:**
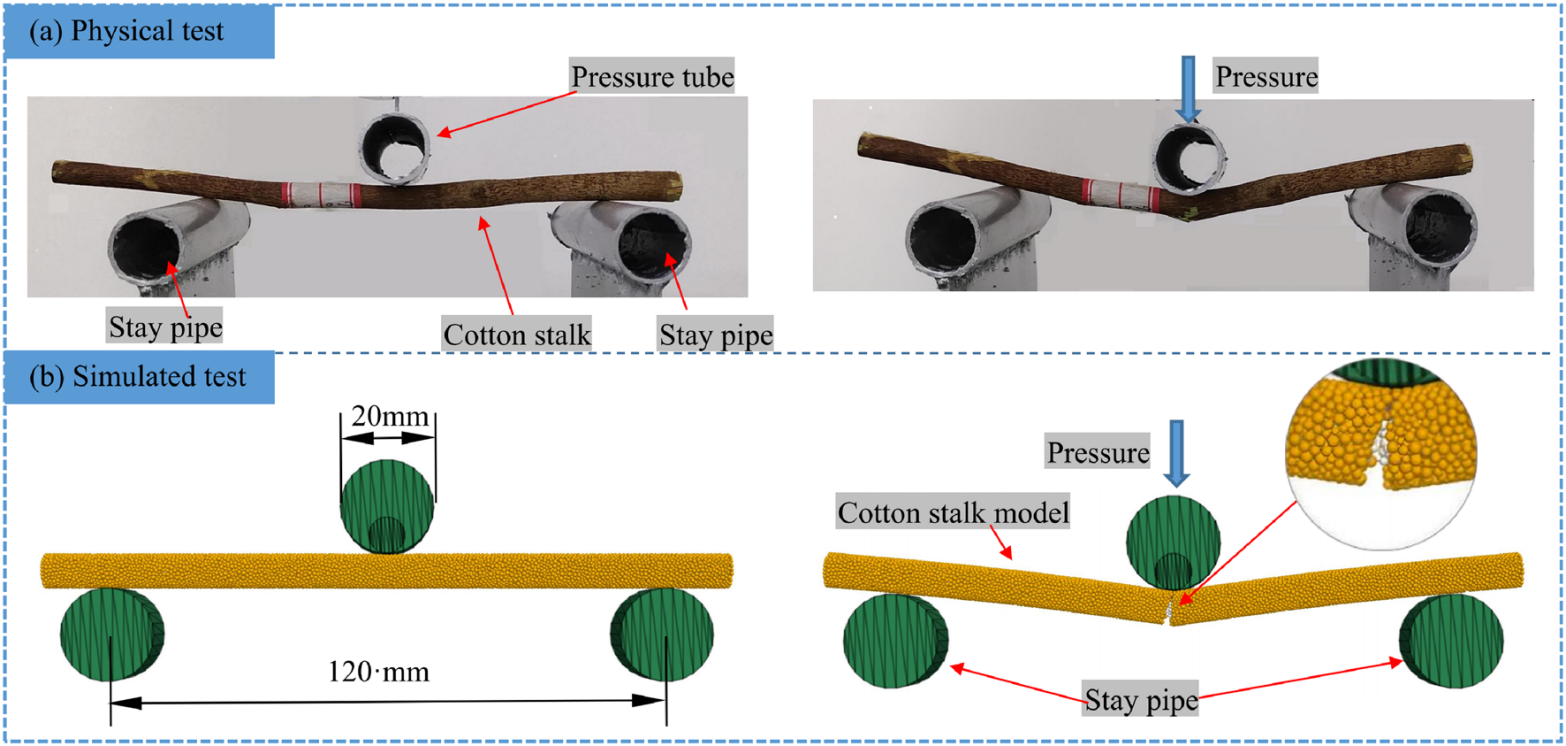
The bending deformation process of the cotton stalk in physical and simulated tests.

The physical and simulated bending deformation processes of cotton stalks are shown in Fig. 11, respectively. The bending process of the cotton stalks is mainly characterized by the elastic compression stage, the compression collapse fracture stage, and the end-yielding stage. In the elastic compression stage, the simulated cotton stalks show elastic behaviors when the applied load is less than the $F_{\rm W}^{max}$. The load is directly proportional to the displacement of the upper compression tube. In the case of continuous feeding of the upper compression tube, the cotton stalks are subjected to a load greater than the $F_{\rm W}^{max}$, which is the stage of crushing. When the $F_{\rm W}^{max}$ is applied, the applied load decreases extremely fast, which is due to the sudden breakage of the cotton stalk, i.e., the stage of compression collapse and fracture. This also proves that the cotton stalk belongs to the brittle material. Finally, the bending force of the cotton stalk shows a fluctuation stage because the contacts at the upper compression tube have not yet completely broken. With the continuous feeding of the upper compression tube, the middle part of the cotton stalk is finally completely disconnected, and the test terminates. The bending deformation processes of the cotton stalk model and the real cotton stalk are consistent.

**Figure 12:**
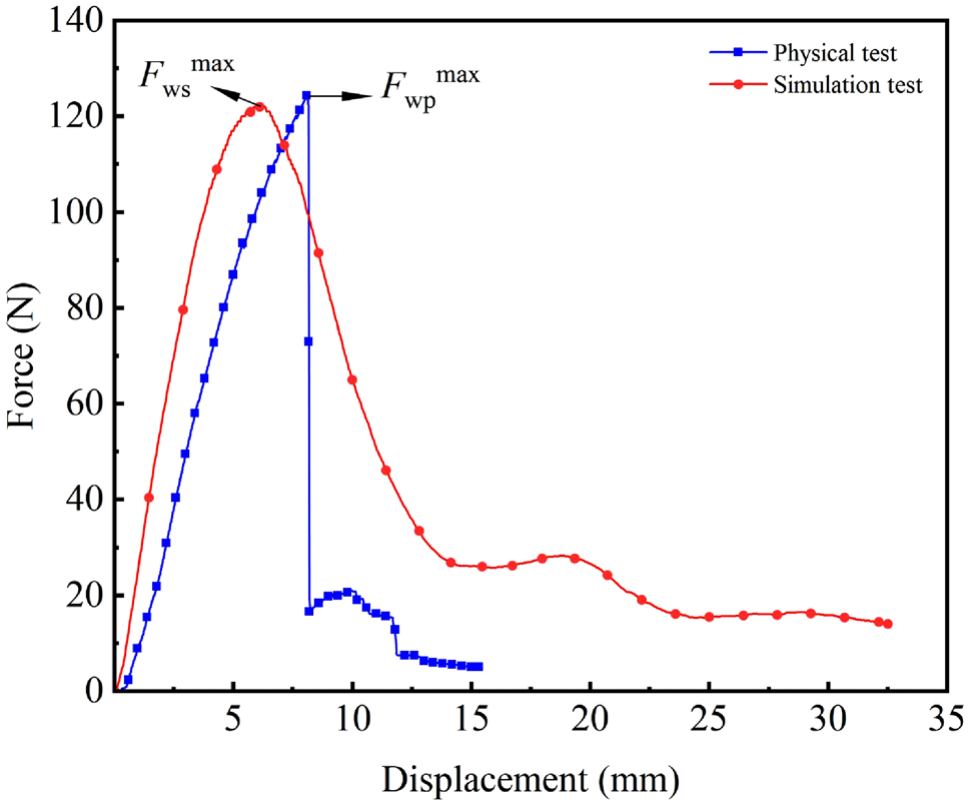
*F*–*x* curve of bending simulation test and physical test.

Figure 12 shows the *F*–*x* curves of the simulated and physical bending tests. The $F_{\rm W}^{max}$ obtained from the simulated test is 123.99 N, and the error relative to the $F_{\rm W}^{max}$ of 120.40 N obtained from the physical test is 2.98%, which indicates that the trends of the two *F*–*x* curves are the same and that the cotton stalks model can simulate the force during the physical bending of the cotton stalks. The different limiting positions of the actual and simulated compressive strengths of the cotton stalks may be due to the large differences in the diameter and wall thickness of the cotton stalk specimens, which provide different deformation positions of the limiting pressure.

#### Analysis and validation of shear tests

To verify the accuracy of the calibration results of the microscopic parameters of the cotton stalks and the shear performance of the simulation model, the discrete element model of cotton stalks with a diameter of 7.50 mm and a length of 150.0 mm is constructed using the same method as that in the simulated compression test. The optimal combination of parameters obtained from the compression test is used for the simulated shear test.

**Figure 13:**
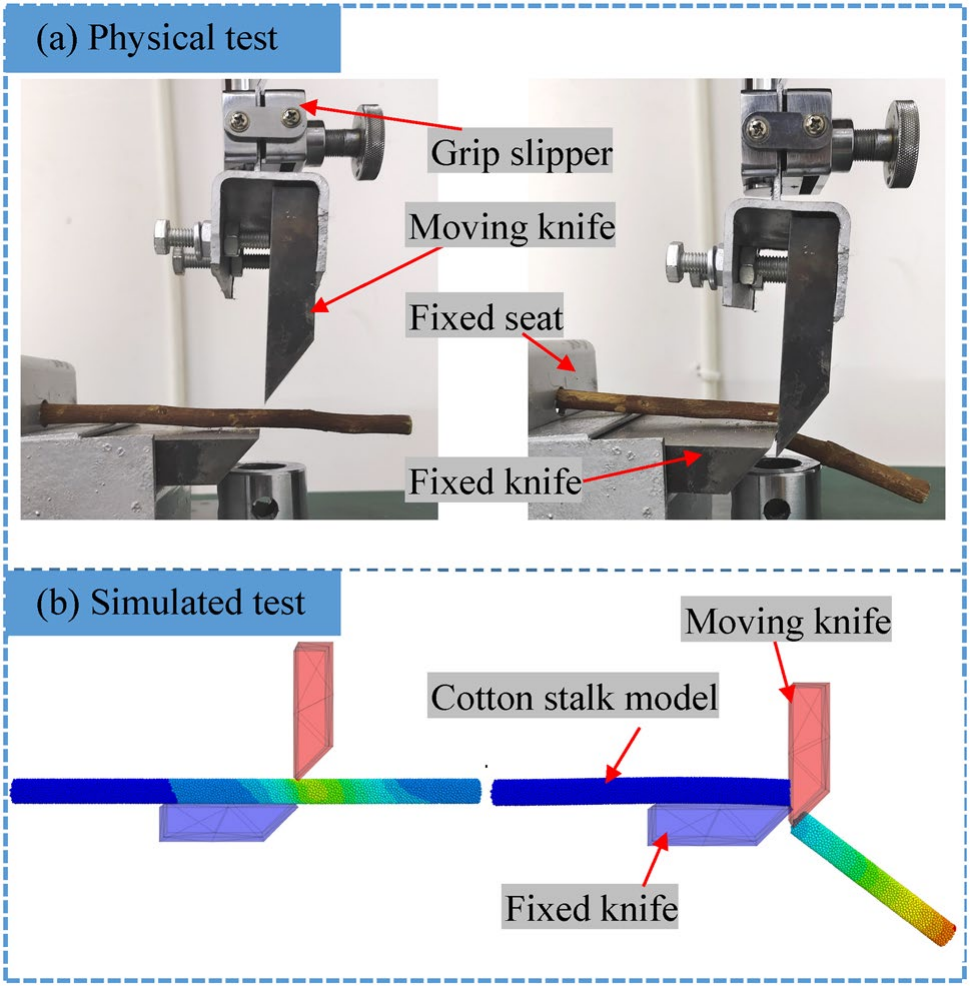
The shear deformation process of the cotton stalk in physical and simulation tests.

Figure 13 shows the deformation process of cotton stalks in physical and simulated shear tests. During the shearing process, with the downward movement of the moving knife, the cotton stalk is deformed under pressure. The cotton stalks on the left and right sides of the blade of the moving knife split, and then the cotton stalks are cut. The simulation model has a high degree of similarity with the real stalk in the deformation process, thus reflecting the fracture and shear behavior of the cotton stalks.

**Figure 14:**
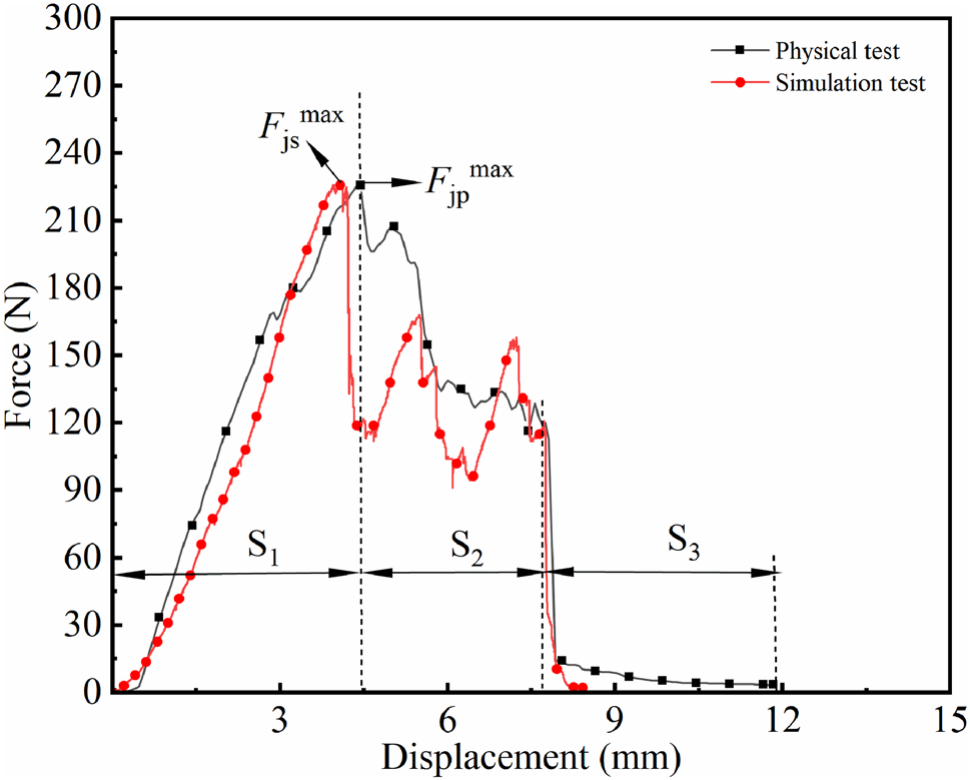
*F*–*x* curve of shear simulation and physical tests.

Figure 14 shows the *F*–*x* curves of the simulated and physical tests. The $F_{\rm j}^{\max }$ obtained from the simulated test is 227.4 N, and the error is 2.52% relative to the $F_{\rm j}^{\max }$ of 221.80 N obtained from the physical test. The established model can simulate the force during the physical shear of the cotton stalks. In the $S_{\rm 1}$ and $S_{\rm 3}$ segments, the trends of the *F*–*x* curves of the two tests are the same, and in the $S_{\rm 2}$ segment, the *F*–*x* curves of the two tests are slightly different due to the unstable mechanical state of the medullary core of the cotton stalks and the fluctuation of *F*–*x* curves during shearing.

In summary, the cotton stalk model can accurately reflect the mechanical properties of real cotton stalks from both macroscopic and microscopic aspects and can be applied to simulate the deformation of stalks and cotton stalks in most cases.

## Conclusions

1. The tensile, compression, bending, and shear tests are carried out on the cotton stalks of the film-stalk mixture by the universal material testing machine. Based on the analysis of the test results, the $F_{\rm l}^{\max }$, $F_{\rm y}^{\max }$, $F_{\rm W}^{\max }$, and $F_{\rm j}^{\max }$ of the cotton stalks, and the $\sigma _{\rm l}^{max}$, $\sigma _{\rm y}^{\max }$, $\sigma _{\rm w}^{\max }$, and $\sigma _{\rm j}^{\max }$, as well as the change rule of the *F*–*x* curves of the cotton stalks under the different loading modes are obtained, and the change rule of the cotton stalks under different loading modes are investigated.
2. A discrete element simulation model of cotton stalks based on a flat-joint bonding model is established with PFC$^{\rm 3D}$ software, and then radial compression simulation tests are carried out. The measured $F_{\rm y}^{\max }$ and *F*–*x* curves are used as the test response values. The law of the significant influence of 13 influence factors on the $F_{\rm y}^{\max }$ is obtained through the *PBD* test, and the significant factors influencing the peak pressure $F_{\rm y}^{\max }$ are screened out. The steepest climb test is performed to determine the optimal range of the significant influence factors. Finally, a regression model with four significant factors is developed using the *BBD* test with the tested $F_{\rm y}^{\max }$ as the target. The regression model is solved and the optimal parameter combinations affecting the peak pressure $F_{\rm y}^{max}$ are obtained as follows: *E*(x-x) is 0.85$\times$10$^8$ Pa, $\sigma _{\rm n}$(x-x) is 6.0525$\times$10$^6$ Pa, $\sigma _{\rm s}$(x-x) is 2.11$\times$10$^7$Pa, $\sigma _{\rm s}$(x-p) is 1.31$\times$10$^7$ mm, $K_{n\mathrm b}$ is 1.0$\times$10$^7$ N/m, $K_{W}$ is 1.0$\times$108 N/m, $k_{\rm g}$ is 1.5, *E*(p-p) is 1.0$\times$10$^6$ Pa, $\sigma _{\rm n}$(p-p) is 5.0$\times$10$^4$ Pa, $\sigma _{\rm s}$(p-p) is 1.0$\times$10$^5$ Pa, *E*(x-p) is 5.0$\times$10$^8$ Pa, $\sigma _{\rm n}$(x-p) is 5.0$\times$10$^7$ Pa, and $\mu _{\rm c}$ is 0.5.
3. The optimal combination of the parameters in the discrete element model of cotton stalks are subjected to compression validation test, three-point bending validation test, and shear validation test by using PFC6.0$^{\rm 3D}$, and the corresponding *F*–*x* curves are extracted. The $F_{\rm y}^{\max }$ obtained from the simulated compression test is 244.11 N, with an error of 0.14% relative to the $F_{\rm ya}^{\max }$ of 244.70 N obtained from the physical compression test. The $F_{\rm W}^{\max }$ obtained from the simulated bending test is 123.99 N, with an error of 2.98% relative to the $F_{\rm Wa}^{\max }$ of 120.40 N obtained from the physical bending test. The $F_{\rm j}^{\max }$ obtained from the simulated shear test is 227.4 N, with an error of 2.52% relative to the $F_{\rm j}^{\max }$ of 221.80 N obtained from the physical bending test. All the *F*–*x* curves are similar, indicating that the damage characteristics of cotton stalks during the simulated and physical tests are consistent the method of parameter selection and calibration is reasonable, and the developed discrete element model can accurately characterize the biomechanical properties of cotton stalks.

## Data Availability

The datasets used and/or analyzed during the current study available from the corresponding author on reasonable request.
